# Comprehensive analysis of prognostic value and immune infiltration of IAPs family in hepatocellular carcinoma

**DOI:** 10.7150/jca.87590

**Published:** 2023-09-11

**Authors:** Xuejie Yang, Xiaoqian Yu, Hui Nie, Wenying Jiang, Jianhua Zhou, Chunlin Ou, Xiaoyun He

**Affiliations:** 1 Department of Pathology, Xiangya Hospital, Central South University, Changsha 410008, Hunan, China.; 2 Department of Oncology, Xiangya Hospital, Central South University, Changsha 410008, Hunan, China.; 3 Departments of Ultrasound Imaging, Xiangya Hospital, Central South University, Changsha 410008, Hunan, China.; 4 National Clinical Research Center for Geriatric Disorders, Xiangya Hospital, Central South University, Changsha, China.

**Keywords:** IAPs family, hepatocellular carcinoma, prognosis, methylation, immune cells, biomarkers, therapeutic target

## Abstract

Hepatocellular carcinoma (HCC) is a malignant tumor with high morbidity and mortality rates. The inhibitors of apoptosis (IAP) family act as oncogenes in various tumor types; however, their functions in HCC remain unclear. Here, we used integrated bioinformatics analysis and experimental verification to assess the expression and the prognostic and clinical value of the IAP family in HCC. Using the University of Alabama at Birmingham Cancer Data Analysis Portal (UALCAN) and the Tumor Immune Estimation Resource (TIMER), we analyzed the expression profiles of IAP family members in HCC tissue, normal tissues, and in patients with different stages and grades of HCC. We further verified the expression level of BIRC2 in 25 HCC samples and matched adjacent normal tissues using quantitative real-time PCR (qRT-PCR), and analyzed its correlation with the marker gene of T-helper type 1 cells (Th1)—STAT1. Meanwhile, the association between BIRC2 and the immunotherapeutic response or immunomodulators was confirmed using the Biomarker Exploration of Solid Tumors (BEST) database. The results showed that NAIP, BIRC2, BIRC3, XIAP, BIRC5, and BIRC6 mRNAs were overexpressed in HCC. The clinical stages, pathological grades, and other clinicopathological features of HCC were closely related to the expression levels of the IAP family members, especially the BIRC2 and BIRC5, which were found to be potential prognostic biomarkers for HCC. Expression of the IAPs was strongly associated with immune cell infiltration. Based on the infiltrative status of various immune cells, HCC patients with high BIRC2 and BIRC5 expression demonstrated poor overall survival (OS) rates. In patients with HCC, BIRC2 expression was noticeably elevated. Concurrently, the expression levels of BIRC2 and STAT1 showed a favorable correlation. BEST database analysis revealed that BIRC2 was a negative predictor of responsiveness to anti-programmed cell death ligand 1 (PD-L1)/cytotoxic T-lymphocyte-associated antigen-4 (CTLA-4) inhibitor treatment in HCC, and BIRC2 mRNA expression levels were positively correlated with the expression levels of the immune checkpoint genes programmed cell death protein 1 (PD-1), PD-L1, and CTLA-4 in HCC. Consequently, the IAP family may play a role in carcinogenesis and cancer-immune system interactions in HCC. Our results demonstrate that IAP family members may be viable predictive biomarkers and therapeutic targets for HCC.

## Introduction

Hepatocellular carcinoma (HCC) is one of the most prevalent malignancies worldwide 1, 2. Patients often have a poor prognosis due to the tumor heterogeneity and distant metastasis of HCC. Over the last two decades, significant advancements have been made in the diagnostic modalities and standard therapies for HCC; however, its recurrence and death rates remain high 3. Hence, new treatments and prognostic biomarkers are urgently needed to improve the survival rates of patients with HCC.

The inhibitors of apoptosis (IAP) family is made up of a highly conserved class of endogenous caspase inhibitors, present in Drosophila to vertebrates. There are eight proteins in this family: BIRC1 (also called NAIP), BIRC2, BIRC3, XIAP, BIRC5, BIRC6, BIRC7, and BIRC8. A defining characteristic of the IAP family is the presence of one or more baculoviral IAP repeats 4. IAPs have a variety of biological functions, including the regulation of apoptosis, innate immunity, inflammation, cell migration, and proliferation 5. IAPs are among the most thoroughly investigated molecular and therapeutic targets for cancer treatment, and dysregulation in their expression has been reported in some malignancies. Studies have shown that the IAP family members are crucial for the occurrence and development of many tumors, such as colorectal cancer (CRC) 6, renal cell carcinoma (RCC) 7, glioma 8, and cervical squamous cell carcinoma (SCC) 9. The underlying molecular mechanisms of the IAP family in the progression of HCC have yet to be fully elucidated. In this study, we used research databases and bioinformatic tools to assess the mRNA expression levels of the IAP family members in HCC patient samples and analyzed their prognostic values, which may be useful for improving treatment outcomes for HCC patients.

## Materials and Methods

### The Cancer Genome Atlas (TCGA) HCC samples

The RNA-seq data and clinicopathological parameters of the HCC cohort sample were acquired from TCGA database 10. HCC samples with missing information in the TCGA dataset were excluded from the study, resulting in a final selection of 342 HCC samples for analysis. To group the HCC samples, we categorized them based on the mean expression of IAPs, creating two distinct groups: one with high expression and another with low expression. Subsequently, by linking the TCGA data to the corresponding mRNA expression values, we conducted an investigation into the relevance of the IAP gene family concerning clinicopathological parameters in the cohort of 342 HCC patients.

### Exclusion and inclusion criteria

Exclusion and inclusion criteria Eligible patients included in this article are in accordance with the following inclusion criteria: (1) pathological confirmation of the diagnosis; (2) prior to resection, none of the patients had received any type of therapy, including chemotherapy, radiation, or immunotherapy; (3) complete clinicopathological data. The detailed clinic parameters of enrolled patients were presented in Table. Exclusion criteria included the following: (1) other treatments were used after the operation; (2) vital organ dysfunction; (3) other organ tumors.

### Patients' tissue samples

A total of 25 pairs of matched adjacent normal tissue samples and paraffin-embedded archival HCC specimens were collected from Xiangya Hospital (Changsha, P. R. China). The HCC tumor specimens were obtained after surgical resection within the period of June 2022 to March 2023. A total of 25 cases were included in this study, comprising 18 male patients and 7 female patients. The age of the patients ranged from 40 to 76 years, with a median age of 60 years. Among the cases, 14 had tumors smaller than 4cm, while 11 cases had tumors measuring 4cm or larger. Tumor grading revealed 5 cases with high differentiation, 16 cases with moderate differentiation, and 3 cases with low differentiation. 23 patients were diagnosed with neural invasion, all 25 patients showed no signs of neural invasion. Moreover, microvascular invasion (MVI) was present in 2 patients, while 13 patients did not exhibit any MVI. The collection of clinical HCC specimens was conducted with the necessary approval from the Research Ethics Board of Xiangya Hospital of Central South University.

### Quantitative real-time polymerase chain reaction

RNA isolation and amplification and quantitative real-time polymerase chain reaction (qRT-PCR) were performed as described previously 11, 12. The thermocycling program used was as follows: 95°C for 30 sec, followed by 40 cycles of 60°C for 30 sec and 72°C for 30 sec. Primer sequences for qRT-PCR are showed in **Table 1**.

### TIMER

We utilized TIMER (https://cistrome.shinyapps.io/timer/) as it provides standardized cancer data sets that facilitate gene expression and survival analysis investigations^13^. We conducted an examination of the mRNA expression levels for IAP family members across multiple cancers using the TIMER database. To convert the data to logarithmic scale, we applied log_2_ transformations with a per million transcripts normalization method. In addition to this, using the TIMER database, we carried out an analysis focusing on the correlation between IAP family members and immune cell infiltration in the tumor microenvironment.

### UALCAN

Users of the UALCAN (http://ualcan.path.uab.edu/) can compare data on mRNA expression in human malignant tumors based on the TCGA dataset 14. To investigate the associations between 371 HCC samples and 50 normal liver samples' clinicopathological characteristics and different IAPs' mRNA expression, we employed the "individual cancer stages" model and "tumor grade" model. To determine the statistical significance of our findings, we conducted a Student's *t*-test, and results with *P*-values less than 0.05 were deemed significant.

### cBioPortal

cBioPortal platform (http://cbioportal.org/) serves as an open, comprehensive resource for the analysis of extensive cancer genomics and clinical data sets 15. In our study, we utilized cBioPortal to examine the genomic profiles of IAP family members in a dataset consisting of 178 HCC samples containing information on mutations and mRNA expression. We imposed a threshold of |log_2_ fold-change (FC)| set at 1, and established a* P*-value cutoff of 0.05 for our analyses.

### STRING

The STRING database (https://cn.string-db.org/) was used to evaluate the associations between various IAP genes and their interactions.

### Cytoscape

We compiled a list of 122 functionally related, co-expressed IAP family members chosen through cBioPortal analysis. Subsequently, we performed a protein-protein interaction (PPI) analysis utilizing the Cytoscape software. The size of each node in the PPI network was determined based on the degree values, which represent the connectivity between interacting proteins.

### Metascape

Metscape (http://metascape.org) is a free tool for gene annotation and analysis that supports a wide range of enrichment analysis techniques and aids researchers in understanding multiple gene lists 16. In our study, we employed this resource to perform Gene ontology (GO) and Kyoto Encyclopedia of Genes and Genomes (KEGG) pathway enrichment analyses for gain insights into the functional characteristics and biological pathways associated with IAPs family members.

### Kaplan-Meier database

Utilizing the Kaplan-Meier database (https://kmplot.com), we analyzed the prognostic significance of the IAP family in HCC 17. The mRNA expression values were utilized to determine the optimal cutoff, thus separating the HCC samples into groups with high and low expression. By linking the TCGA data to the corresponding mRNA expression values using the plotter, we investigated the prognostic relevance of the IAP gene family in HCC patients.

### TISIDB

The Tumor and Immune System Interaction Database (TISIDB) (http://cis.hku.hk/TISIDB) is an online resource designed to explore the connections between tumors and the immune system, incorporating a diverse range of data sources. In our study, we used the TISIDB to examine if the expression of IAPs varied among different immune subtypes of HCC patients.

### BEST

Biomarker Exploration of Solid Tumors (BEST) portal (https://rookieutopia.com/app_direct/BEST/) was used for validation the association between BIRC2 and immunomodulators, as well as immunotherapeutic response 18.

### Statistical analysis

SPSS (version 26.0) was used to conduct statistical analysis on the relationships between the mRNA expression of IAPs and the clinicopathological characteristics of patients with HCC. Student's *t*-tests were used for comparisons and *P-*values less than 0.05 were considered statistically significant.

## RESULTS

### Aberrant expression of members of the IAP family in HCC patients

**Figure 1:**
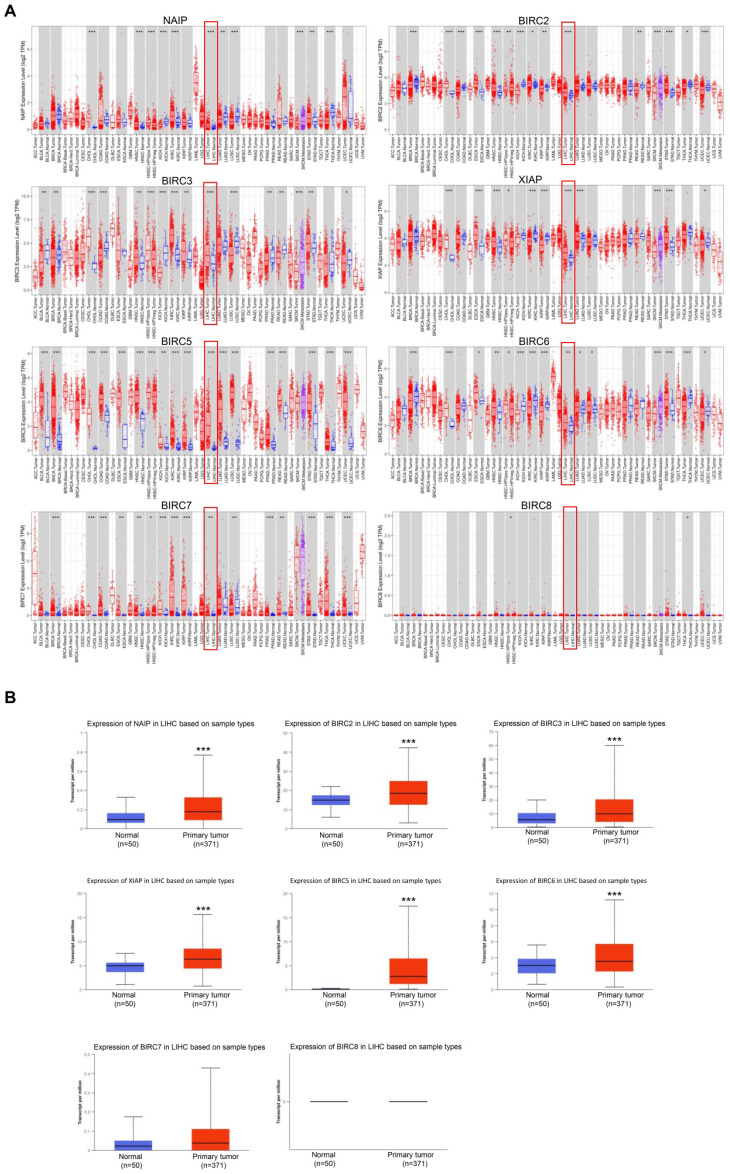
** The inhibitors of apoptosis (IAP) gene family is overexpressed in hepatocellular carcinoma (HCC). (A)** The mRNA expression levels of IAP family members across various cancers were detected by TIMER. **(B)** The relative expression of the IAP family in HCC. Statistical significance is indicated as * *P* < 0.05, ** P < 0.01, *** P < 0.001 compared to the control group.

In our study, we utilized the TIMER database to examine the mRNA expression levels of the IAP family across various tumor and normal tissues. The analysis revealed that several members of the IAP family, including NAIP, BIRC2, BIRC3, XIAP, BIRC5, BIRC6, and BIRC7, exhibited higher expression levels in multiple cancers, including HCC (**Figure 1A**). However, the expression of BIRC8 did not show statistical significance. To further investigate the expression patterns and clinical significance of IAPs in HCC, we analyzed IAP expression in tumor and normal liver tissues using the UALCAN database. The results indicated that NAIP, BIRC2, BIRC3, XIAP, BIRC5, and BIRC6 showed significantly higher expression levels in tumor tissues than in normal tissues (**Figure 1B**). This observation suggests that IAPs may contribute to tumor progression in HCC.

### Association between the mRNA levels of IAP family members and the clinicopathological features of patients with HCC

**Figure 2:**
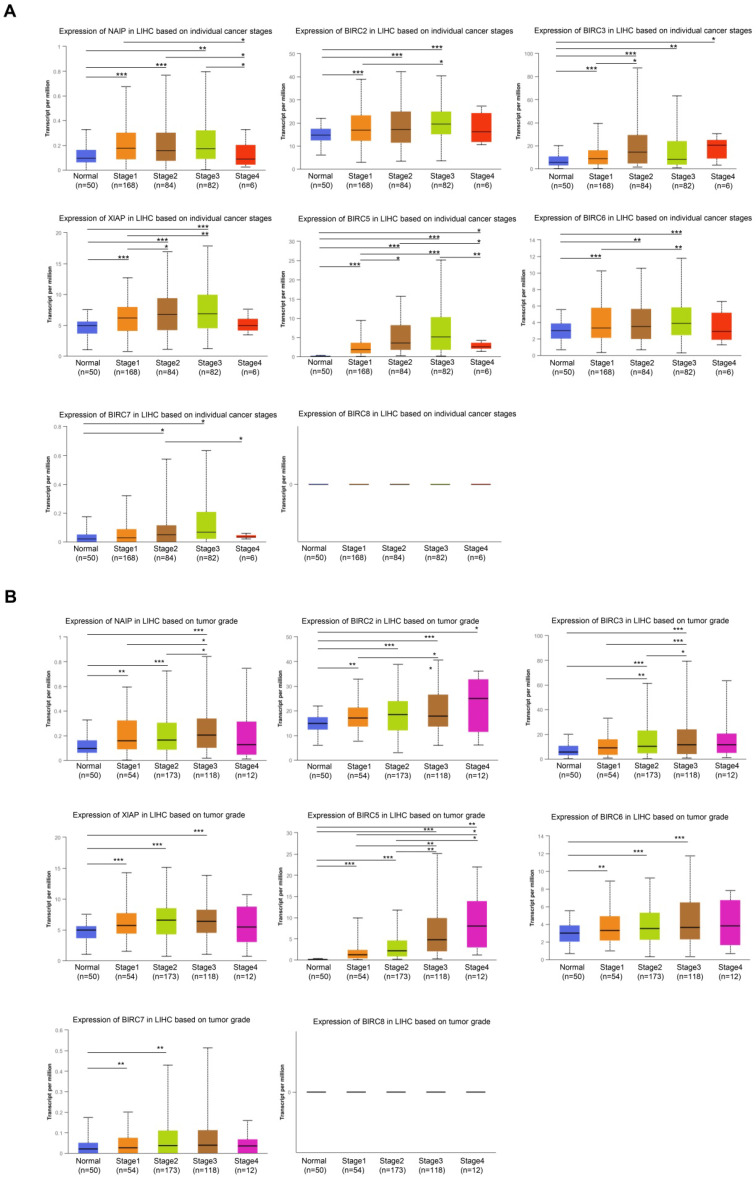
** The association between the mRNA expression of various IAP family members and the clinicopathological characteristics of HCC patients was examined using UALCAN. (A)** The correlation between the mRNA expression of different IAP family members and the pathological stage of HCC in patients. **(B)** The relationship between tumor grade in HCC patients and the mRNA expression of IAP family members. Statistical significance is indicated as * P < 0.05, ** P < 0.01, *** P < 0.001 compared to the control group.

The expression of IAPs in relation to two crucial clinicopathological parameters, the tumor stage and grade of HCC, was further examined using patient samples from the UALCAN database. Notable changes were observed in the expression levels of BIRC3 and BIRC5 across different tumor stages. The levels of NAIP, BIRC2, XIAP, and BIRC6 were significantly elevated in the subgroups of tumor stages 1-3 compared to their levels in the normal subgroups (**Figure 2A**), with no discernible differences in their levels in the subgroup of tumor stage 4. BIRC2 and BIRC5 expression gradually and significantly increased from grades 1 to 4. The mRNA expression levels of NIAP, XIAP, and BIRC6 tended to be higher in grades 1 to 3 (**Figure 2B**).

Furthermore, we expanded our analysis by including 342 HCC patient samples from the TCGA database to investigate the correlations between the IAP gene family and the pathological characteristics of HCC (**Table 2**). The results revealed significant associations between the expression of IAP family members and various clinicopathological parameters in HCC patients. We observed that high levels of NIAP, BIRC5, BIRC6, and BIRC8 expression were significantly associated with the T stage of HCC patients. The expression of NIAP and BIRC6 showed a correlation with sex, while NIAP and BIRC5/6/7/8 expression demonstrated associations with the pathological stage of HCC. Additionally, high expression levels of all eight IAP members were significantly associated with the histological grade of HCC. These findings suggest that the differentiation of tumor cells may, to some extent, indicate the potential diagnostic value of IAP family members in HCC. The identified correlations between IAP expression and the clinicopathological parameters of HCC provide valuable insights into the potential role of IAPs as diagnostic markers for HCC.

### Genetic alterations and functional enrichment analysis of members of the IAP family

**Figure 3:**
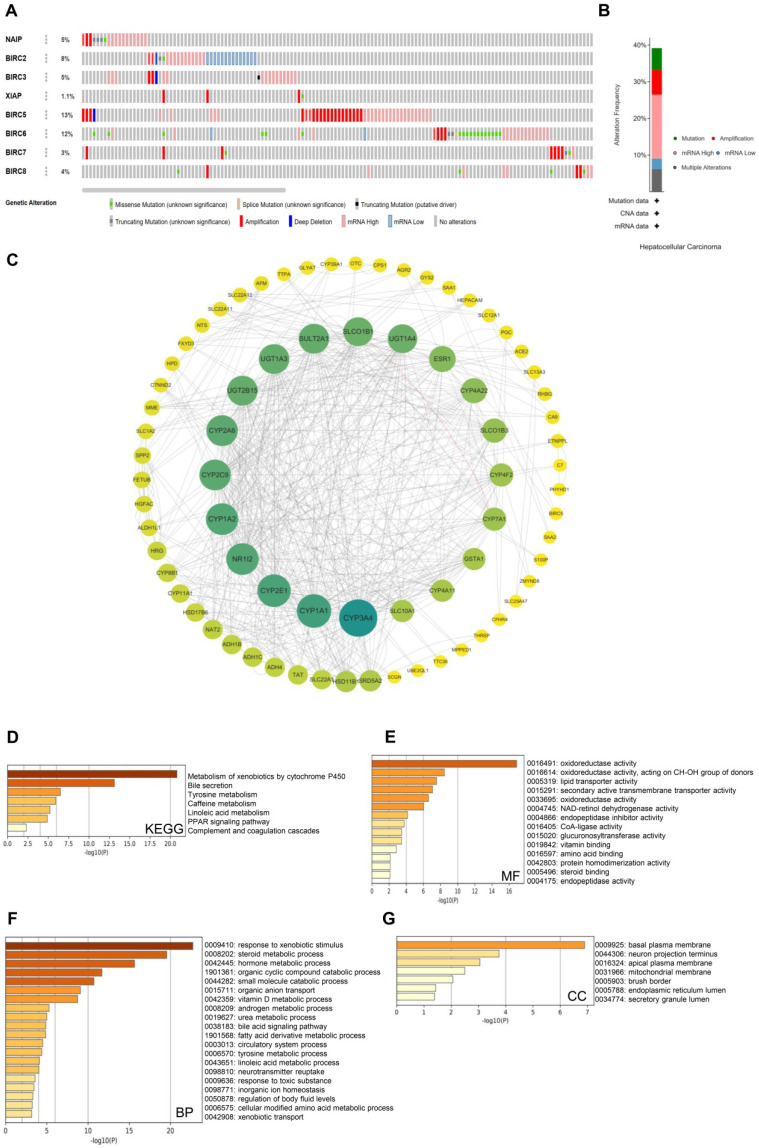
** Genetic alterations and enrichment analysis of IAPs in HCC. (A-B)** An overview of the mutation rates of IAPs in HCC cases. **(C)** A protein-protein interaction (PPI) network involving members of the IAP family and their partners was constructed using cBioPortal and Cytoscape.** (D-G)** KEGG enrichment pathway analysis examined molecular functions, biological processes, and cellular components related to the co-expressed genes of IAPs.

Genetic alterations are widely recognized as crucial factors in cancer development. To investigate the genetic alteration status of the IAP family members, we utilized the cBioPortal database for comprehensive analysis. Out of 178 HCC patient samples, alterations in IAP genes were observed in 80 samples (45%), with BIRC5 displaying the highest alteration rate (13%). The most common abnormalities within the IAP family were mRNA alterations, followed by amplifications and other mutations (**Figure 3A and 3B**). To delve deeper into the co-expression networks linked with the IAP family members, we identified co-expressed genes using the cBioPortal database with a threshold of |log_2_FC| ≥ 1 and *P*-value < 0.05. The resulting network of key genes related to the IAP family was visualized using Cytoscape_v.3.9.0 (**Figure 3C**). Notably, CYP3A4, CYP1A1, CYP2E1, CYP1A2, NR1I2, CYP2C9, CYP2A6, and UGT2B15 were identified to be primarily associated with the modulation and function of IAPs in HCC.

In addition, we employed the Metascape database to perform GO and KEGG pathway analyses on the co-expressed genes of IAP family members in HCC patient samples. The KEGG pathway analysis highlighted key pathways such as the metabolism of xenobiotics by cytochrome P450 enzymes, bile secretion, tyrosine metabolism, and complement and coagulation cascades (**Figure 3D**). Molecular function analysis revealed their involvement in oxidoreductase activity, specifically acting on the CH-OH group of donors, as well as lipid transporter activity (**Figure 3E**). Biological process analysis indicated their role in responding to xenobiotic stimuli, steroid metabolic processes, hormone metabolic processes, and organic cyclic compound and small molecule catabolic processes (**Figure 3F**). Cellular component analysis unveiled their frequent association with the basal plasma membrane, neuron projection terminus, apical plasma membrane, and mitochondrial membrane (**Figure 3G**). These results suggest that the IAP family may participate in drug metabolism and complement and coagulation cascades in HCC patients, potentially influencing their treatment outcomes and immune infiltration.

### Association between the expression levels of IAPs and immune infiltration in HCC

**Figure 4:**
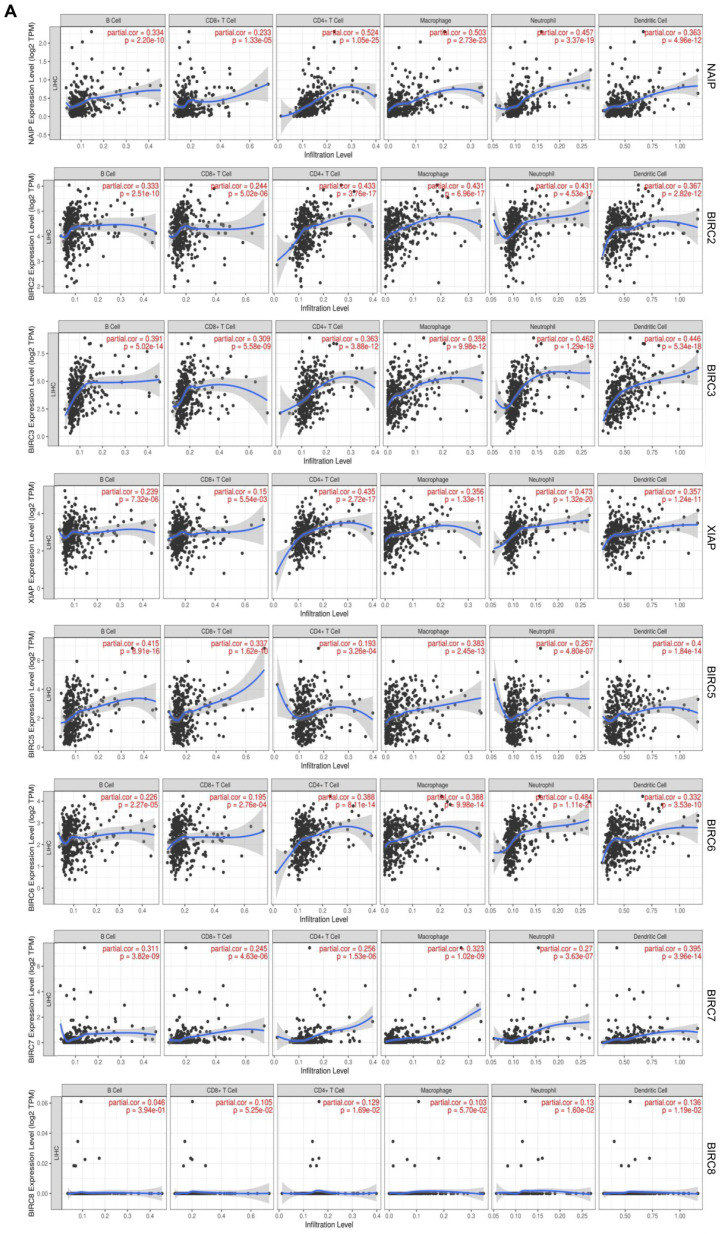
** Association between the expression of IAPs and immune infiltration in HCC. (A)** The effect of IAP family members on the immune cell infiltration of HCC was analyzed by TIMER.

To comprehend the functions of the IAP family members in HCC, we employed TIMER to investigate the molecular characteristics of tumor-immune interactions.** Figure 4** illustrates that except of BIRC8, the transcriptional expression of the remaining IAPs exhibited a positive correlation with the expression of B cells, CD8+ T cells, CD4+ T cells, neutrophils, macrophages, and dendritic cells (DCs) (R> 0.1, *P* < 0.05). This evidence implies that members of the IAP family might influence the immune response within the tumor microenvironment of HCC.

Our study elucidated the associations between IAPs and the signature marker genes of various immune cells in HCC (**Table 3**). We observed a strong correlation between the expression of NAIP and the expression of several immune cells, including CD8+ T cells, M1 and M2 macrophages, tumor-associated macrophages (TAMs), DCs, natural killer cells (NKs), B cells, T-helper type 1 cells (Th1), Th2, neutrophils, regulatory T cells (Tregs), and monocytes. A close correlation was also observed between the expression of BIRC2 and the expression of B cells, neutrophils, TAM, M1 and M2 macrophages, Tfh, Th1, Th2, Tregs, Th17, DCs, and monocytes. A moderate correlation was identified between the expression of XIAP and the expression of neutrophils, TAM, Th1, Th2, DCs, and Treg markers. Correlations in expression levels were moderately close between BIRC5 and neutrophils, Th1, Th2, CD8+ T cells, TAM, B cells, and monocytes. In terms of expression levels, BIRC6 exhibited very strong correlations with DCs, TAMs, M1 and M2 macrophages, Th1, Th2, Tregs, neutrophils, and monocyte markers in HCC patients. BIRC7, on the other hand, showed relatively high correlations with TAMs, M1, B cells, Tfh, T cells, and Th17 cells. These findings suggest that IAP family members play pivotal roles in immune infiltration in HCC.

### Prognostic value of mRNA expression of IAPs in HCC patients

**Figure 5:**
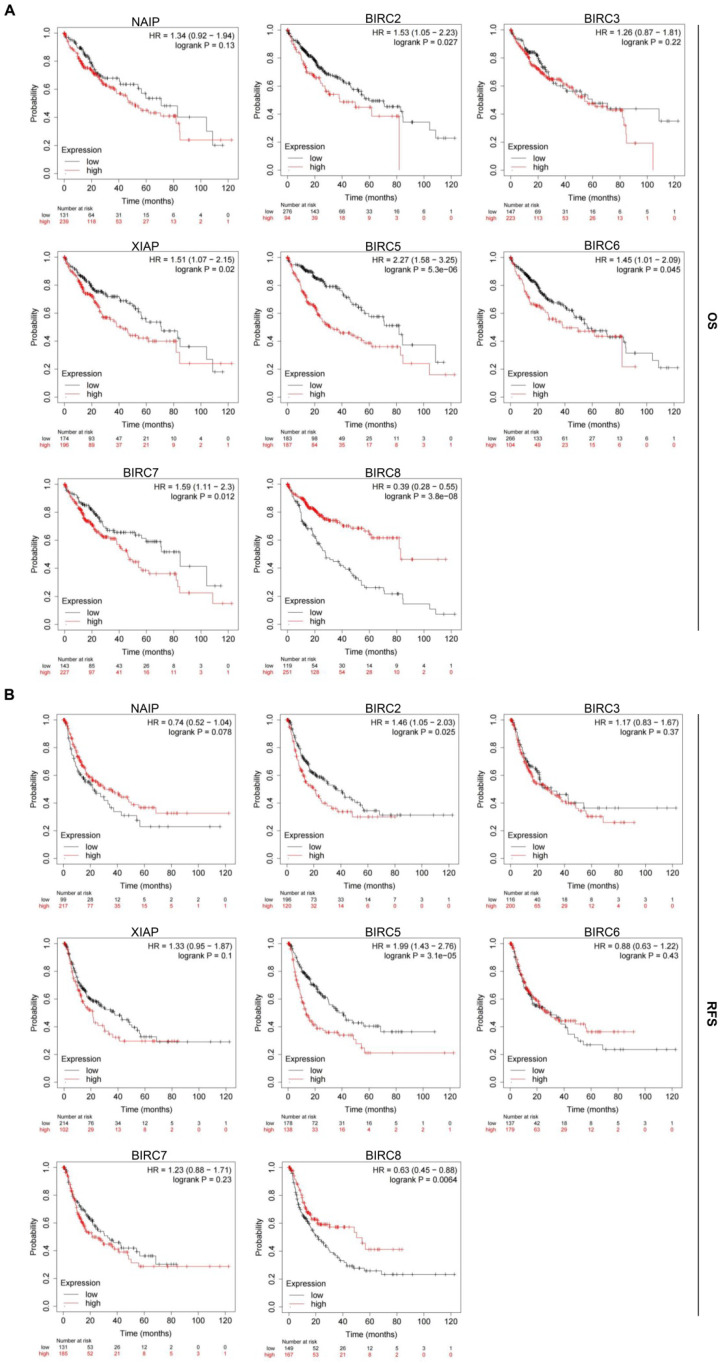
** Prognostic significance of the IAP family in HCC. (A-B)** The relationship between mRNA expression of IAP family members and overall survival (OS) and progression-free survival (RFS) rates in HCC patients was examined using the Kaplan-Meier plotter.

To assess the prognostic value of the mRNA expression of IAP family members in HCC patients, we utilized the Kaplan-Meier plotter to analyze both overall survival (OS) and relapse-free survival (RFS) rates. We found that elevated expression of BIRC2, XIAP, BIRC5, BIRC6, and BIRC7 was significantly associated with poor OS rates (*P* < 0.05;** Figure 5A**). Furthermore, patients with higher transcription levels of BIRC2 and BIRC5 experienced shorter RFS rates (*P* < 0.05; **Figure 5B**). Patients with higher transcript levels of BIRC8 had longer OS and RFS rates (*P* < 0.05). Based on these findings, we proposed that BIRC2 and BIRC5 may potentially serve as prognostic biomarkers for HCC patients.

**Figure 6:**
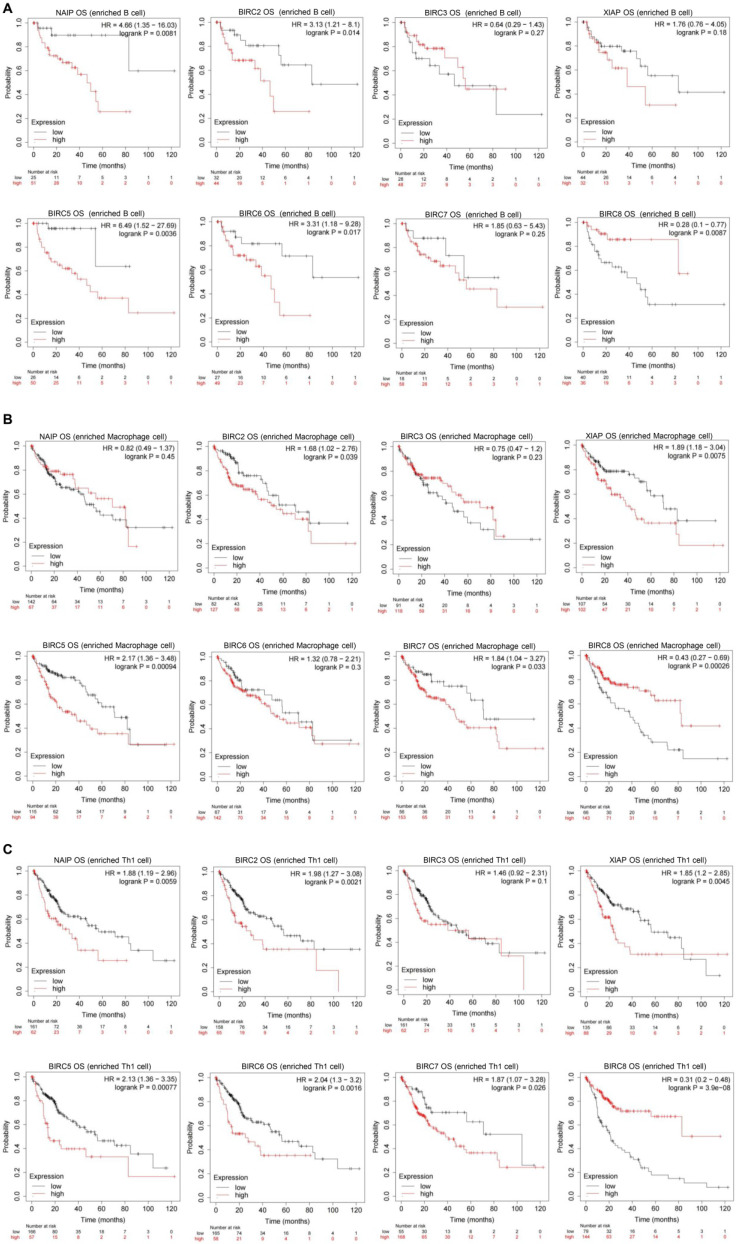
** The mRNA gene expression levels of the IAP family members in HCC are correlated with the overall survival (OS) rates of HCC patients.** Associations between mRNA gene expression levels of IAPs and the OS rates of HCC patients who had (A) B cells, (B) macrophages, and (C) Th1 cells enriched.

We conducted further prognostic analysis using the Kaplan-Meier plotter, focusing on the expression levels of IAPs in HCC patients within the relevant immune cell subgroups (**Figure 6**). The findings revealed that upregulation of BIRC2 and BIRC5 was correlated with poor OS rates and RFS rates when patients with HCC were enriched with B cells, macrophages, and Th1 cells. In patients with B cell enrichment, NAIP and BIRC6 expression showed a significant negative correlation with OS rates, and high levels of XIAP and BIRC7 were associated with shorter OS rates when HCC patients were enriched with macrophages. Similarly, high NAIP, XIAP, BIRC6, and BIRC7 expression in the enriched Th1 cells cohort was associated with a worse prognosis. The above analyses indicate that high expression of IAPs in HCC may affect the prognosis due in part to immune infiltration of HCC.

### Relationship between BIRC2 expression in HCC and tumor immune subtypes and immunotherapy

**Figure 7:**
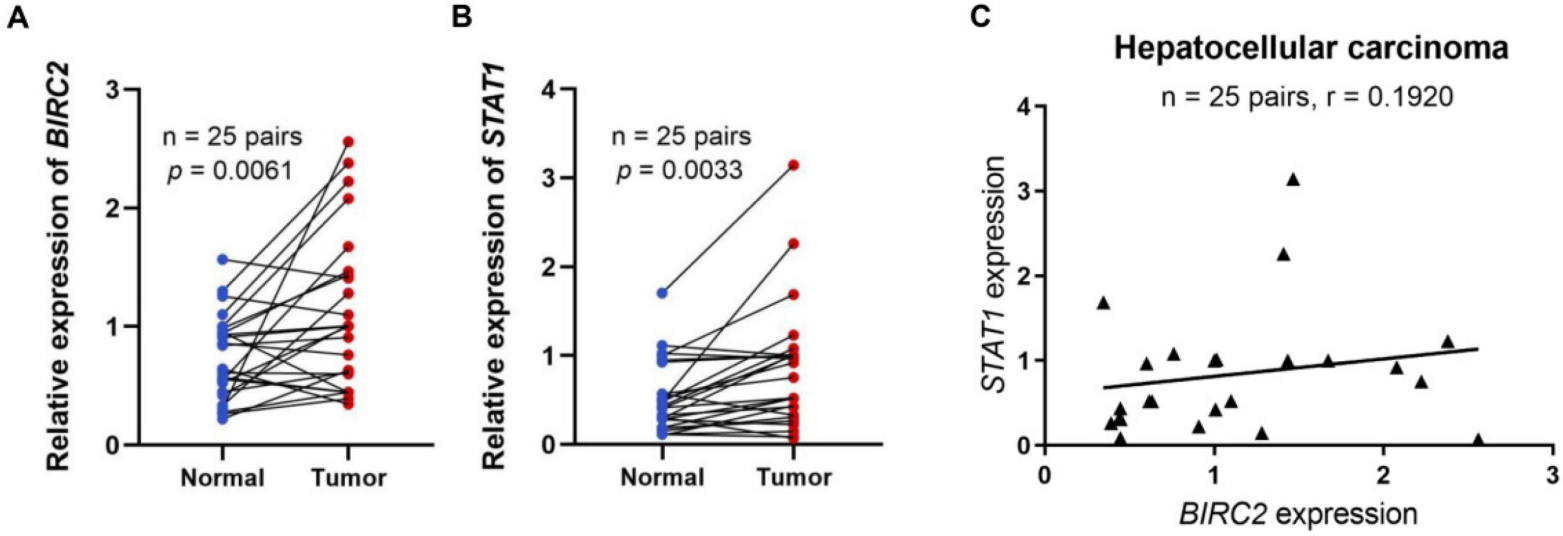
** The mRNA expression levels of BIRC2 and STAT1 in HCC tissues. (A)** The mRNA expression levels of BIRC2 in HCC. **(B)** The mRNA expression levels of STAT1 in HCC tissues. **(C)** The relationships between the mRNA expression levels of BIRC2 and STAT1 in HCC tissues.

Based on the above analysis, we found that BIRC2 may play an important role in promoting the progression of HCC. To further explore the role of BIRC2 in HCC, we verified the differential expression of BIRC2 in HCC tissues and its correlation with immune cell-associated molecules using qRT-PCR. The results revealed that the relative expression levels of BIRC2 in the 25 HCC tissue samples were significantly higher than the BIRC2 expression levels in the matched adjacent non-tumor tissue samples (*P* < 0.05; **Figure 7A**​). Moreover, we demonstrated a relationship between BIRC2 and STAT1 19. Using qRT-PCR analysis, we found that the relative expression levels of STAT1 were significantly higher in the 25 HCC tissues than the expression of STAT1 in the matched paracancerous normal tissues (*P* < 0.05, **Figure 7B**). After comparing the expression of BIRC2 and STAT1 in the 25 HCC samples, we found a weak positive correlation (R= 0.1920; **Figure 7C**). Moreover, we further analyzed the relationship between the expression levels of BIRC2 or STAT1 and the clinicopathological characteristics of HCC patients, and found that the expression levels of BIRC2 or STAT1 were significantly associated with the differentiation degree of HCC in patients (*P* < 0.05, **Table 4**).

**Figure 8:**
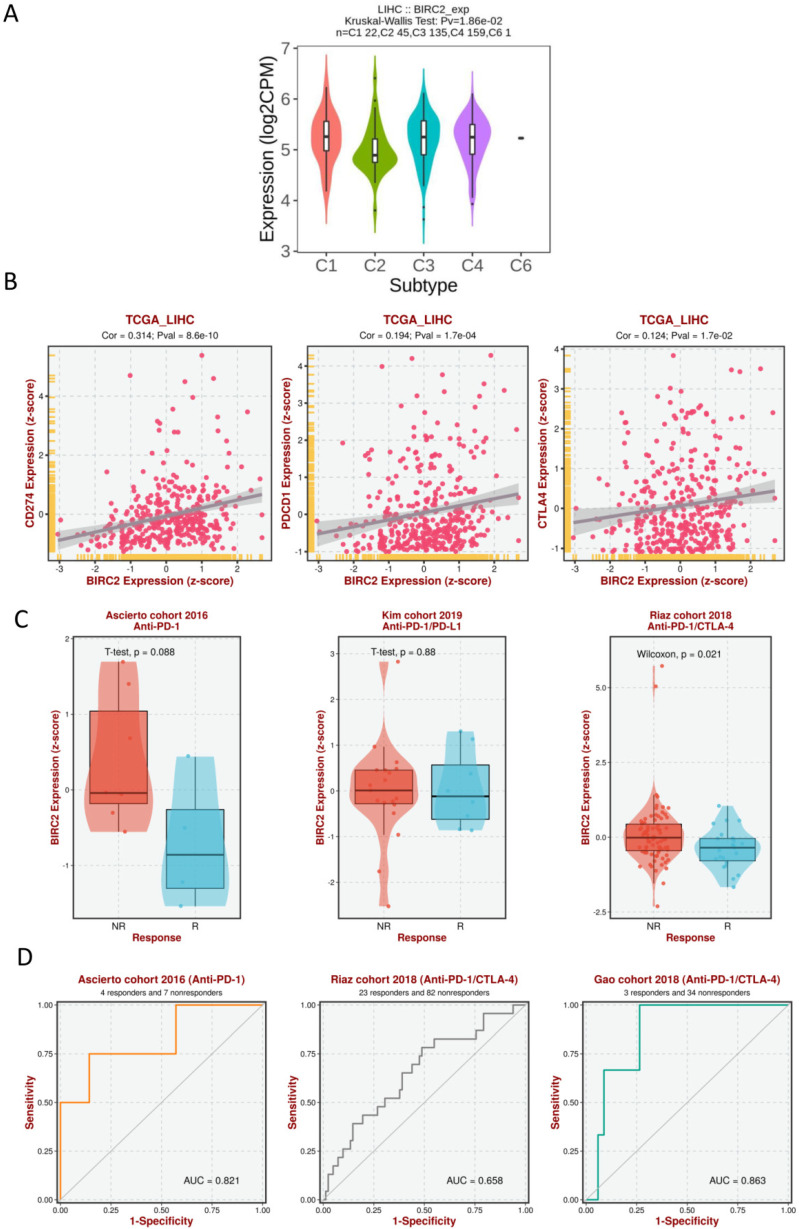
** The association of BIRC2 mRNA expression and the immunotherapeutic response.** (A) The relationship between BIRC2 mRNA expression and the different immune subtypes of HCC (TISIDB). (B) The correlation between the expression of immune checkpoint genes PDCD1 (Programmed cell death protein 1, PD-1), CD274 (Programmed cell death protein 1, PD-L1), and cytotoxic T-lymphocyte-associated antigen-4 (CTLA-4) with the mRNA expression of BIRC2 in HCC tissue (BEST). (C) A comparison in the mRNA expression of BIRC2 in anti-PD-1, anti-PD-1/PD-L1, and anti-PD-1/CTLA-4 responders and nonresponders based on the Asicerto, Kim, and Riaz cohorts (BEST). (D) ROC curve of BIRC2 expression for patients in the Asicerto, Riaz, and Gao cohorts (BEST).

We investigated whether the expression of IAPs differed between different immune subtypes of HCC (**Figure 8A**). The results showed that BIRC2 expression differed significantly between the four immune subtypes (C1, C2, C3, and C4), indicating that BIRC2 plays a vital role in the immune infiltration of HCC. To further test the correlation between BIRC2 and immunotherapy, the association of the expression of known immune checkpoint genes 20 with BIRC2 in HCC tissue was analyzed using BEST. As shown in **Figure 8B**, a positive correlation was observed between the mRNA expression levels of BIRC2 and those of PDCD1 (programmed cell death protein 1, PD-1), CD274 (programmed death ligand 1, PD-L1), and cytotoxic T-lymphocyte-associated antigen-4 (CTLA-4). Moreover, using BEST analysis, we wanted to determine whether aberrant BIRC2 expression could affect the immunotherapeutic response to HCC. As shown in **Figure 8C**, the expression of BIRC2 was elevated in anti-PD-1 nonresponders in the Ascierto cohort and in the anti-PD-1/PD-L1 nonresponders in the Kim cohort. Similarly, anti-PD-1/CTLA-4 nonresponders in the Riaz cohort expressed BIRC2 mRNA in abundance. The area under the receiver operating characteristic curve values for the Ascierto, Riaz, and Gao cohorts were 0.821, 0.658, and 0.863, respectively, indicating that BIRC2 can distinguish between anti-PD-1 and anti-PD-1/CTLA-4 responders and nonresponders (**Figure 8D**).

## Discussion

IAP family members are frequently overexpressed and associated with poor prognosis in human cancers. Lin et al. demonstrated that miR-143-3p sensitized the response of α7-HPV-related cervical SCC to chemotherapy by targeting BIRC2, an IAP family member associated with the poor prognosis of many cancers. This indicates that BIRC2 may be used as a novel prognostic factor and therapeutic target 9. Moreover, another IAP family member, XIAP regulates cancer initiation, promotion, and progression. Several drugs targeting XIAP are currently under development, mainly containing small-molecule antagonists (SMAC mimetics) and antisense oligonucleotides (AEG35156) 21. BIRC5 expression is significantly higher in cancer tissue than in normal tissue in 16 different cancer types 22, and several studies have implicated BIRC5 in chemoresistance to platinum- or taxane-based chemotherapy in ovarian cancer 23, 24. Additionally, BIRC6 is believed to play an important role in the progression and chemoresistance of several cancers 25-27. Increased BIRC6 expression in non-small cell lung cancer (NSCLC) is linked to an advanced pathological T stage, poor differentiation, and lymph node metastasis, and may be associated with tumor progression. Therefore, targeting BIRC6 may be useful for the treatment of NSCLC 28. Although the roles of IAPs in tumor development and progression have been partially confirmed, the specific roles of the IAP family members in HCC remain to be determined. This is the first study to comprehensively explore the mRNA expression levels, prognostic values, functional enrichment, and immunotherapeutic relationships of IAPs in HCC. We explored the differential mRNA expression of each IAP family member in HCC tissue compared to their expression in normal tissue using the TIMER and UALCAN databases. NAIP, BIRC2, BIRC3, XIAP, BIRC5, and BIRC6 mRNAs were overexpressed in HCC cells compared to their expression within normal cells. Another novel finding was that the IAPs were closely associated with the individual clinicopathological stages, tumor grades, and T stage of HCC. These data suggest that IAPs may be associated with HCC progression and that members of the IAP family may act as potential diagnostic markers for HCC. Moreover, the IAP family members exhibited frequent genetic alterations during HCC. Alterations in mRNA expression are among the most frequent mutations. These findings strongly imply that the differential expression of IAP family members may be essential for HCC progression.

Next, we explored the molecular and biological functions of the members of the IAP family, for their capacity to metabolize xenobiotics and other foreign entities as they are eliminated from the body. Cytochrome P450 and other families of drug-metabolizing enzymes were commonly considered and investigated 29. KEGG pathway analysis of IAP family members and their co-expressing genes revealed that the metabolism of xenobiotics through the cytochrome P450 pathways was significantly enriched, indicating that the members of the IAP family may serve as therapeutic drug targets for HCC. Meanwhile, according to KEGG pathway analyses, complement and coagulation cascades were especially associated with IAPs. The three main outcomes of complement activation are the opsonization of pathogens, recruitment of inflammatory and immune-competent cells, and direct pathogen killing 30. Our results suggest that IAP-associated signaling is essential for antitumor immunity as it influences the recruitment of immunocompetent cells.

Increasing evidence suggests that immune cell infiltration may significantly affect tumor development and recurrence, and may play a significant role in determining the effectiveness of immunotherapy and clinical outcomes 31, 32. We discovered a significant relationship between the mRNA expression of IAPs and the infiltration of six different immune cell types: B cells, CD8+ T cells, CD4+ T cells, macrophages, neutrophils, and DCs. Therefore, we investigated the relationship between the mRNA expression of IAPs and the mRNA expression of immune infiltration markers in patients with HCC; various immune cells demonstrated significant relationships with the mRNA expression of IAPs. Many studies have also found that immune cell infiltration is closely associated with OS 33, 34. Therefore, we investigated the association between the mRNA expression of IAP family members and the prognosis of patients with HCC. Elevated expression levels of BIRC2 and BIRC5 were associated with worse OS and RFS rates in patients with HCC. When HCC patients were enriched with B cells, macrophages, and Th1 cells, overexpression of BIRC2 and BIRC5 was associated with poor OS rates. These findings imply that BIRC2 and BIRC5 are important oncogenes in HCC. Furthermore, the expression of BIRC2 was significantly correlated with the expression of the Th1 cell marker gene STAT1 and the prognosis of HCC patients enriched with Th1 cells. To further confirm the differential expression of BIRC2 in HCC and its association with immune cells, we collected 25 pairs of paraffin-embedded archived HCC specimens to detect BIRC2 expression and matched them against the BIRC2 expression in adjacent normal tissue samples. BIRC2 expression was considerably upregulated in patients with HCC. BIRC2 expression positively correlated with the expression of the signature marker gene of Th1 cells, STAT1.

BIRC2, a member of the IAP family, also known as cIAP1, serves as a therapeutic target for chemotherapy against various tumors. Zhen et al. discovered that AT406, an IAP antagonist, induced degradation of IAPs (cIAP-1 and XIAP) and exhibited cytotoxic and pro-apoptotic effects on HepG2, SMMC-7721 cell lines, and primary HCC cells 35. Similarly, Tian et al. found that the small-molecule IAP inhibitor LCL16 targeted cIAP1, cIAP2, and XIAP, which are upregulated in HCC tumors, suggesting that LCL161 may be an effective agent in combination with paclitaxel for treating liver tumors 36. The first tyrosine kinase inhibitor, sorafenib, was the only drug approved for first-line treatment of HCC. Experimental evidence suggests that sorafenib weakens the translation of c-IAP1 mRNA by targeting its internal ribosome entry site. Additionally, ectopic expression of c-IAP1 alleviates sorafenib-induced cancer cell apoptosis 37. BIRC2 plays an important role in tumor chemotherapy, and it can also be used as a target for tumor immunotherapy. Cancer immunotherapy, which was recently approved for HCC, has emerged as an effective treatment strategy that targets the immune system 38. However, tumor immunotherapy is effective only in a small number of individuals with HCC and other solid tumors. Therefore, it is crucial to understand the composition of various immune cell types in the tumor microenvironment and their interactions with the tumor cells. Recent trials have demonstrated that the development of new therapeutic options has improved the survival of patients with HCC 39. The response rate to immune checkpoint inhibitors (ICIs) or targeted drugs is low. Thus, a combination of different ICIs or immunotherapies with other treatments (targeted and locoregional therapies) is effective for HCC. Cytotoxic T-lymphocyte-associated antigen-4 (CTLA-4) is a B7/CD28 family member and inhibits T cell function. Ipilimumab, an anti-CTLA-4 blocking antibody, was the first immune checkpoint inhibitor to be tested and has been approved for treating cancer patients. The most widely used immunotherapeutic drugs are PD-1/PD-L1 checkpoint inhibitors. They are antibodies against the membrane receptors PD-1 and PD-L1 and control cell migration, proliferation, and secretion of cytotoxic mediators 40, 41. A combination of the anti-PD-1 antibody nivolumab and the anti-CTLA-4 antibody ipilimumab has been approved for treating melanoma, RCC, CRC with high microsatellite instability, and non-small cell lung cancer 42. In the area of HCC, nivolumab plus ipilimumab has been granted accelerated approval by the FDA as a second-line therapy after sorafenib 43, and a phase 3 trial using this combination of drugs is currently underway. Atezolizumab is a humanized monoclonal antibody against PD-L1. The combination of atezolizumab and bevacizumab is currently the preferred first-line therapy for patients with HCC who are not at risk of bleeding 44. In October 2020, the tremelimumab (a CTLA-4 inhibitor) plus durvalumab (a PD-L1 inhibitor) regimen was the first combination immunotherapy with anti-PD-L1 and anti-CTLA-4 antibodies approved for the treatment of adult patients with unresectable HCC 45. Samanta et al. demonstrated BIRC2-mediated immune evasion and immune checkpoint blockade (ICB) resistance in cancer cells 46. They discovered that mouse melanoma and breast tumors were significantly more sensitive to anti-CTLA4 and/or anti-PD1 ICB when there was a deficiency in the expression of BIRC2. In this study, we aimed to determine whether aberrant BIRC2 expression influences the immunotherapeutic response to HCC. We found that BIRC2 is a negative marker for anti-PD-L1/CTLA4 inhibitor therapy in HCC. Our results support the immunosuppressive functions of BIRC2 by demonstrating a strong association between the expression of BIRC2 and the expression of immunological molecules in HCC, which indicates that BIRC2 plays a crucial role in tumor immunity and is a promising biomarker for predicting the prognosis and effectiveness of immunotherapy in patients with HCC.

In summary, this is the first study to analyze the relationship between the mRNA expression of IAPs and tumor immune infiltration in HCC, which may provide a better comprehension on the crucial functions of these genes in the development of tumors and in the immune system of HCC patients. Moreover, we identified the potential of IAPs as useful biomarkers and therapeutic targets that can be used to develop diagnostic and prognostic approaches to improve treatment outcomes for HCC patients. However, this study had significant limitations that need to be considered. To validate the probable processes behind the actions of multiple IAP family members in HCC, as well as the molecular connections between them and the clinical applications of these genes, more studies are required, including both* in vitro* and clinical studies.

## Supplementary Material

Supplementary table.

## Figures and Tables

**Table 1:** Primer sequence for qRT-PCR

Gene	Primer (Forward)	Primer (Reverse)
BIRC2	AGCACGATCTTGTCAGATTGG	GGCGGGGAAAGTTGAATATGTA
STAT1	CGGCTGAATTTCGGCACCT	CAGTAACGATGAGAGGACCCT
U6	CTCGCTTCGGCAGCACA	AACGCTTCACGAATTTGCGT

**Table 2:** Relationship between clinicopathologic parameters and IAP family members expression in RCC

Characteristics	N	NAIP				BIRC2				BIRC3				XIAP		
		Low	High	*P*		Low	High	*P*		Low	High	*P*		Low	High	*P*
**Gender**																
Male	233	169	64	**0.011**		116	117	0.454		169	64	0.852		137	96	0.759
Female	109	64	45			59	50			78	31			66	43	
**Age (year)**																
≤60	170	119	51	0.460		85	85	0.667		122	48	0.920		103	67	0.645
>60	172	114	58			90	82			125	47			100	72	
**T stage**																
T1 + T2	256	167	89	**0.048**		129	127	0.619		186	70	0.757		153	103	0.791
T3 + T4	86	66	20			46	40			61	25			50	36	
**N stage**																
Nx	87	60	27	0.846		42	45	0.532		61	26	0.611		49	38	0.504
N0 + N1	255	173	82			133	122			186	69			154	101	
**M stage**																
Mx	77	49	28	0.337		37	40	0.534		58	19	0.490		38	39	**0.045**
M0 + M1	265	184	81			138	127			189	76			167	102	
**Pathologic stage**																
Stage I + II	252	164	88	**0.043**		127	125	0.632		183	69	0.784		151	101	0.243
Stage III + IV	90	69	21			48	42			64	26			52	46	
**Histologic grade**																
grade 1 + 2	213	147	66	**0.000**		105	108	**0.000**		163	50	**0.000**		121	92	**0.000**
grade 3 + 4	129	86	43			70	59			84	45			82	47	
**Characteristics**	**N**	** BIRC5**				** BIRC6**				** BIRC7**				** BIRC8**		
		**Low**	** High**	***P***		**Low**	** High**	***P***		**Low**	** High**	***P***		**Low**	** High**	***P***
**Gender**																
Male	233	155	78	0.361		138	95	**0.031**		212	21	0.415		225	8	0.209
Female	109	67	42			51	58			102	7			102	7	
**Age (year)**																
≤60	170	104	66	0.66		89	81	**0.000**		158	12	0.449		161	9	0.415
>60	172	118	54			100	72			156	16			166	6	
**T stage**																
T1 + T2	256	179	77	**0.001**		143	113	**0.000**		235	21	0.985		248	8	**0.049**
T3 + T4	86	43	43			169	40			79	7			79	7	
**N stage**																
Nx	87	62	25	**0.003**		51	36	0.466		77	10	0.193		80	7	0.372
N0 + N1	255	160	95			138	117			237	18			247	8	
**M stage**																
Mx	77	60	17	**0.007**		42	35	0.886		69	8	0.423		69	8	**0.003**
M0 + M1	265	162	103			147	118			245	20			258	7	
**Pathologic stage**																
Stage I + II	252	176	76	**0.000**		142	110	**0.000**		231	21	**0.000**		244	8	**0.000**
Stage III + IV	90	46	44			47	43			83	7			83	7	
**Histologic grade**																
grade 1 + 2	213	155	58	**0.000**		117	96	**0.000**		201	12	**0.000**		205	8	**0.000**
grade 3 + 4	129	67	62			72	57			113	16			122	7	

Bold font indicates significant difference.

**Table 3:** The relationship between immune cell markers and the expression of IAP family members

		NAIP		BIRC2		BIRC3		XIAP	
		**cor**	***p***	**cor**	***p***	**cor**	***p***	**cor**	***p***
**CD8+ T cell**	CD8A	0.298	***	0.193	***	0.368	***	0.127	*
	CD8B	0.157	**	0.092	0.076	0.294	***	0.009	0.860
	GZMA	0.197	***	0.068	0.188	0.381	***	0.008	0.884
**B cell**	CD19	0.227	***	0.141	***	0.294	***	0.068	0.193
	CD79A	0.272	***	0.106	**	0.309	***	0.028	0.591
	MS4A1	0.297	***	0.144	***	0.328	***	0.079	0.129
**T cell**	CD3D	0.209	***	0.079	0.128	0.407	***	0.011	0.827
	CD3E	0.307	***	0.150	***	0.467	***	0.092	0.076
	CD2	0.301	***	0.127	*	0.462	***	0.071	0.170
**TAM**	CCL2	0.320	***	0.218	***	0.403	***	0.216	***
	CD68	0.288	***	0.148	***	0.340	***	0.204	***
	IL10	0.404	***	0.263	***	0.378	***	0.230	***
**M1**	IRF5	0.484	***	0.394	***	0.317	***	0.359	***
	PTGS2	0.438	***	0.312	***	0.445	***	0.330	***
	NOS2	0.208	***	0.188	***	0.075	0.149	0.244	***
**M2**	MS4A4A	0.339	***	0.201	***	0.470	***	0.272	***
	CD163	0.339	***	0.239	***	0.381	***	0.292	***
	VSIG4	0.334	***	0.202	***	0.456	***	0.276	***
**Neutrophils**	ITGAM	0.388	***	0.276	***	0.582	***	0.362	***
	CCR7	0.382	***	0.190	***	0.378	***	0.167	**
	SIGLEC5	0.464	***	0.391	***	0.438	***	0.389	***
**DC**	HLA-DQB1	0.232	***	0.098	0.059	0.391	***	0.099	0.056
	HLA-DPB1	0.294	***	0.177	***	0.484	***	0.195	***
	HLA-DRA	0.320	***	0.239	***	0.553	***	0.288	***
	HLA-DPA1	0.349	***	0.244	***	0.516	***	0.289	***
	ITGAX	0.505	***	0.326	***	0.512	***	0.293	***
	CD1C	0.343	***	0.256	***	0.339	***	0.185	***
	NRP1	0.433	***	0.530	***	0.250	***	0.564	***
**NK cell**	KIR2DL1	0.050	0.333	-0.001	0.981	-0.001	0.977	0.065	0.209
	KIR2DL3	0.177	***	0.181	***	0.179	***	0.164	**
	KIR2DL4	0.171	***	0.103	*	0.263	***	0.109	*
	KIR3DL1	0.139	**	0.118	*	0.047	0.369	0.164	**
	KIR3DL2	0.183	***	0.126	*	0.148	**	0.055	0.294
	KIR3DL3	0.103	*	0.071	0.173	0.042	0.422	0.009	0.864
	KIR2DS4	0.195	***	0.115	*	0.064	0.222	0.092	0.076
**Th1**	TBX21	0.248	***	0.129	*	0.292	***	0.104	*
	STAT1	0.487	***	0.484	***	0.522	***	0.398	***
	STAT4	0.412	***	0.234	***	0.523	***	0.162	**
	IFNG	0.196	***	0.110	*	0.292	***	0.093	0.073
**Th2**	STAT6	0.349	***	0.399	***	0.246	***	0.516	***
	GATA3	0.420	***	0.226	***	0.409	***	0.215	***
	STAT5A	0.386	***	0.349	***	0.361	***	0.395	***
	IL13	0.236	***	0.099	0.056	0.138	**	0.118	*
**Tfh**	BCL6	0.294	***	0.434	***	0.089	0.085	0.426	***
	IL21	0.196	***	0.072	0.164	0.120	*	0.077	0.140
**Th17**	STAT3	0.427	***	0.418	***	0.448	***	0.503	***
	IL17A	0.147	**	0.109	*	0.100	0.054	0.084	0.105
**Treg**	FOXP3	0.362	***	0.235	***	0.171	***	0.289	***
	STAT5B	0.470	***	0.643	***	0.129	*	0.621	***
	CCR8	0.548	***	0.386	***	0.466	***	0.424	***
	TGFB1	0.436	***	0.307	***	0.321	***	0.219	***
**T -cell exhaustion**	PDCD1	0.331	***	0.185	***	0.341	***	0.101	0.051
	CTLA4	0.281	***	0.108	*	0.415	***	0.065	0.212
	HAVCR2	0.421	***	0.262	***	0.512	***	0.263	***
	LAG3	0.202	***	0.058	0.267	0.139	**	-0.017	0.748
**Monocyte**	CD86	0.419	***	0.285	***	0.512	***	0.268	***
	C3AR1	0.411	***	0.293	***	0.499	***	0.334	***
	CSF1R	0.375	***	0.242	***	0.489	***	0.284	***
		BIRC5		BIRC6		BIRC7		BIRC8	
		cor	*p*	cor	*p*	cor	*p*	cor	*p*
CD8+ T cell	CD8A	0.144	**	0.124	*	0.321	***	0.038	0.461
	CD8B	0.217	***	-0.010	0.843	0.368	***	0.044	0.397
	GZMA	0.133	*	-0.010	0.845	0.339	***	-0.016	0.753
B cell	CD19	0.229	***	0.059	0.256	0.303	***	0.095	0.068
	CD79A	0.118	*	0.031	0.551	0.330	***	0.060	0.251
	MS4A1	0.001	0.980	0.089	0.087	0.225	***	0.055	0.291
T cell	CD3D	0.324	***	-0.031	0.552	0.454	***	0.118	*
	CD3E	0.153	**	0.075	0.150	0.395	***	0.041	0.427
	CD2	0.185	***	0.049	0.343	0.408	***	0.042	0.420
TAM	CCL2	-0.022	0.674	0.165	**	0.308	***	-0.004	0.946
	CD68	0.141	**	0.170	**	0.458	***	0.047	0.365
	IL10	0.145	**	0.220	***	0.429	***	0.030	0.560
M1	IRF5	0.294	***	0.405	***	0.186	***	0.032	0.545
	PTGS2	-0.028	0.595	0.336	***	0.286	***	0.047	0.369
	NOS2	-0.138	**	0.292	***	-0.081	0.118	0.021	0.681
M2	MS4A4A	0.009	0.862	0.208	***	0.369	***	-0.027	0.603
	CD163	-0.032	0.542	0.289	***	0.291	***	-0.011	0.838
	VSIG4	0.025	0.631	0.182	***	0.297	***	-0.018	0.735
Neutrophils	ITGAM	0.251	***	0.261	***	0.373	***	0.090	0.085
	CCR7	-0.028	0.588	0.166	**	0.283	***	0.001	0.986
	SIGLEC5	0.123	*	0.397	***	0.340	***	0.049	0.343
DC	HLA-DQB1	0.147	**	0.033	0.526	0.378	***	-0.022	0.669
	HLA-DPB1	0.133	*	0.127	*	0.341	***	-0.009	0.858
	HLA-DRA	0.085	0.101	0.207	***	0.295	***	-0.018	0.726
	HLA-DPA1	0.064	0.221	0.223	***	0.329	***	-0.011	0.829
	ITGAX	0.248	***	0.270	***	0.538	***	0.063	0.229
	CD1C	0.016	0.760	0.241	***	0.145	**	0.095	0.066
	NRP1	0.049	0.351	0.585	***	0.178	***	0.039	0.457
NK cell	KIR2DL1	-0.047	0.364	0.072	0.168	0.021	0.688	-0.004	0.939
	KIR2DL3	0.123	*	0.124	*	0.130	*	-0.044	0.400
	KIR2DL4	0.185	***	0.053	0.311	0.224	***	0.047	0.372
	KIR3DL1	-0.039	0.457	0.154	**	0.118	*	-0.051	0.327
	KIR3DL2	0.061	0.241	0.047	0.371	0.139	**	-0.030	0.563
	KIR3DL3	0.042	0.418	0.012	0.822	0.030	0.564	-0.050	0.338
	KIR2DS4	-0.002	0.968	0.118	*	0.081	0.121	-0.096	0.064
Th1	TBX21	-0.005	0.929	0.096	0.065	0.276	***	-0.041	0.429
	STAT1	0.215	***	0.500	***	0.230	***	0.085	0.100
	STAT4	0.207	***	0.190	***	0.278	***	0.030	0.564
	IFNG	0.257	***	0.058	0.269	0.381	***	0.051	0.323
Th2	STAT6	-0.082	0.115	0.496	***	0.013	0.806	-0.019	0.717
	GATA3	0.145	**	0.212	***	0.388	***	0.062	0.236
	STAT5A	0.230	***	0.329	***	0.387	***	0.080	0.125
	IL13	0.055	0.290	0.114	*	0.063	0.226	-0.001	0.981
Tfh	BCL6	-0.018	0.734	0.407	***	0.046	0.373	0.078	0.136
	IL21	0.095	0.067	0.144	**	0.095	0.067	0.058	0.263
Th17	STAT3	-0.047	0.362	0.508	***	0.153	**	0.127	*
	IL17A	-0.006	0.912	0.127	*	0.054	0.303	0.101	0.052
Treg	FOXP3	-0.001	0.991	0.335	***	0.163	**	0.099	0.056
	STAT5B	0.029	0.583	0.739	***	-0.072	0.166	0.104	*
	CCR8	0.188	***	0.447	***	0.332	***	0.130	*
	TGFB1	0.218	***	0.238	***	0.427	***	0.013	0.800
T -cell exhaustion	PDCD1	0.312	***	0.047	0.365	0.421	***	0.070	0.179
	CTLA4	0.355	***	-0.002	0.972	0.484	***	0.079	0.129
	HAVCR2	0.275	***	0.206	***	0.542	***	0.065	0.214
	LAG3	0.344	***	0.013	0.801	0.333	***	0.106	*
Monocyte	CD86	0.248	***	0.224	***	0.476	***	0.035	0.504
	C3AR1	0.139	**	0.272	***	0.431	***	0.019	0.717
	CSF1R	0.119	*	0.208	***	0.387	***	0.003	0.958

Correlation R value was calculated by Spearman's algorithm and adjusted by tumor purity.*P < 0.05, **P< 0.01, *** P < 0.001.

**Table 4:** Relationship between BIRC2 or STAT1 expression and clinicopathologic parameters in HCC

Characteristics	N	BIRC2			STAT1		
		Low	High	*P*	Low	High	*P*
**Gender**							
Male	18	11	7	0.152	11	7	0.152
Female	7	2	5		2	5	
**Age (year)**							
≤60	14	8	6	0.569	7	7	0.821
>60	11	5	6		6	5	
**MVI**							
M0	23	13	10	0.133	13	10	0.133
M1	2	0	2		0	2	
**Differentiation degree**							
Poor	4	1	3	**0.025**	1	3	**0.025**
Moderate	16	9	7		9	7	
Well	5	3	2		3	2	
**Diameter (cm)**							
<4	14	7	7	0.825	6	8	0.312
≥4	11	6	5		7	4	

Bold font indicates significant difference.
